# Zolbetuximab plus chemotherapy in Japanese patients with claudin 18.2–positive gastric or gastroesophageal junction adenocarcinoma: a combined subgroup analysis of the phase 3 SPOTLIGHT and GLOW trials

**DOI:** 10.1007/s10120-026-01738-7

**Published:** 2026-05-07

**Authors:** Kensei Yamaguchi, Hirokazu Shoji, Hisateru Yasui, Eiji Oki, Daisuke Sakai, Tetsuya Hamaguchi, Akihito Tsuji, Takashi Oshima, Masahiro Tsuda, Keiko Minashi, Jianning Yang, Abraham Guerrero, Yoko Ueno, Maria Matsangou, Georgia Gourgioti, Yuka Nakanishi, Satomi Furuki, Kana Kuwamoto, Shunsuke Yamada, Kohei Shitara

**Affiliations:** 1 Cancer Institute Hospital of the Japanese Foundation for Cancer Research, Tokyo, Japan; 2 National Cancer Center Hospital, Tokyo, Japan; 3 Kobe City Medical Center General Hospital, Hyogo, Japan; 4 Kyushu University, Fukuoka, Japan; 5 Osaka University, Osaka, Japan; 6 Saitama Medical University International Medical Center, Saitama, Japan; 7 Kagawa University, Kagawa, Japan; 8 Kanagawa Cancer Center, Kanagawa, Japan; 9 Hyogo Cancer Center, Hyogo, Japan; 10 Chiba Cancer Center, Chiba, Japan; 11 Astellas Pharma Global Development, Inc., Northbrook, IL USA; 12 Astellas Pharma, Inc., Tokyo, Japan; 13 Astellas Pharma Europe Ltd., Addlestone, UK; 14 Department of Gastrointestinal Oncology, National Cancer Center Hospital East, Kashiwa, Japan

**Keywords:** Zolbetuximab, Stomach neoplasms, Esophagogastric junction, Adenocarcinoma, Claudins

## Abstract

**Background:**

Zolbetuximab is a monoclonal antibody targeting claudin 18 isoform 2 (CLDN18.2) approved for first-line treatment of CLDN18.2-positive, human epidermal growth factor receptor 2 (HER2)-negative, locally advanced (LA) unresectable or metastatic gastric or gastroesophageal junction (GEJ) adenocarcinoma. Here, we report efficacy and safety outcomes from the combined Japanese subgroup analysis of SPOTLIGHT and GLOW.

**Methods:**

Global, double-blind, phase 3 SPOTLIGHT (NCT03504397) and GLOW (NCT03653507) trials investigated zolbetuximab plus chemotherapy versus placebo plus chemotherapy in patients with CLDN18.2-positive, HER2-negative, LA unresectable or metastatic gastric or GEJ adenocarcinoma. The primary endpoint in both trials was progression-free survival (PFS); overall survival (OS) was a key secondary endpoint.

**Results:**

Of 1072 patients enrolled, 116 Japanese patients (SPOTLIGHT, n = 65; GLOW, n = 51) comprised the combined Japanese subgroup. The baseline characteristics of Japanese patients were generally similar to those of the overall study populations. In the combined Japanese subgroup, PFS was improved with zolbetuximab plus chemotherapy versus placebo plus chemotherapy (20.53 months [95% confidence interval (CI) 12.09–not estimable] versus 8.28 months [95% CI 6.57–9.07]). Median OS was 23.06 months (95% CI 16.49–25.49) in the zolbetuximab group versus 16.53 months (95% CI 10.38–18.33) in the placebo group. No new safety signals were observed.

**Conclusions:**

In Japanese patients, zolbetuximab plus chemotherapy improved PFS versus placebo plus chemotherapy and showed a numerical improvement in OS. These results support zolbetuximab plus chemotherapy as a potential new standard-of-care first-line option for Japanese patients with CLDN18.2-positive, HER2-negative, LA unresectable or metastatic gastric or GEJ adenocarcinoma.

**Supplementary Information:**

The online version contains supplementary material available at 10.1007/s10120-026-01738-7.

## Introduction

Globally, gastric cancer is the fifth most commonly diagnosed cancer, with the highest region-specific age-standardized incidence rate reported in Eastern Asia [1, 2]. Gastric cancer is often diagnosed at a later stage because many patients do not experience symptoms at earlier stages [2]. Given the high incidence and delays in diagnosis, radiographic screening programs for gastric cancer were implemented in Japan in the 1960s [3]. More recently, an update to the Japanese Guidelines for Gastric Cancer Screening recommended either radiographic or endoscopic gastric cancer screenings for population-based screening for gastric cancer [3]. However, among patients diagnosed with advanced-stage gastric cancer, the 5-year survival rate is less than 30%, highlighting the unmet need in this population [2].

The treatment of gastric cancer with targeted therapies such as human epidermal growth factor receptor 2 (HER2)-targeted therapies or immune checkpoint inhibitors in combination with chemotherapy has demonstrated significant benefits in survival over treatment with chemotherapy alone [4, 5]. Despite the availability of targeted therapies, chemotherapy continues to be a mainstay of treatment in first-line unresectable advanced or metastatic gastric cancer [6, 7]. With the approval of zolbetuximab for the treatment of patients with claudin 18 isoform 2 (CLDN18.2)-positive, HER2-negative, unresectable, advanced or recurrent gastric cancer, an additional targeted therapy is now available [8–10].

CLDN18.2 is a tight junction protein expressed in gastric epithelial cells but absent in most other healthy tissue [11, 12]. CLDN18.2 is retained in many gastric and gastroesophageal junction (GEJ) adenocarcinomas [11, 13]. Loss of cell polarity during malignant transformation may cause CLDN18.2 to become exposed on the surface of gastric cancer cells, increasing its accessibility to therapeutic antibodies [12]. Among patients with HER2-negative, locally advanced (LA) unresectable or metastatic gastric or GEJ adenocarcinoma, the prevalence of CLDN18.2 positivity (defined as  ≥75% of tumor cells demonstrating moderate to strong membranous CLDN18 staining) was found to be 38.4% in a global patient population [13]. Although the sample size is limited (N = 71), prevalence of CLDN18.2 positivity in Japanese patients in a phase 1 trial was 35.2%, similar to that reported in the global population [14].

Zolbetuximab is a first-in-class immunoglobulin G1 monoclonal antibody that targets and binds to CLDN18.2 [11, 15]. Upon binding, zolbetuximab activates both antibody-dependent cellular cytotoxicity and complement-dependent cytotoxicity [15, 16]. Zolbetuximab was first approved, prior to any other global approval, in Japan in March 2024 for use in combination with antitumor or anticancer drugs for patients with CLDN18.2-positive, HER2-negative, unresectable, advanced or recurrent gastric cancer [8], making it the first and only approved therapy targeting CLDN18.2. Additional global approvals have followed, notably in Europe, the United States, and China, among other geographies [9, 10, 17]. All approvals were based on positive results from the SPOTLIGHT (NCT03504397) and GLOW (NCT03653507) trials. Both SPOTLIGHT and GLOW were global, double-blind, randomized, phase 3 trials investigating zolbetuximab plus chemotherapy for first-line treatment of patients with CLDN18.2-positive, HER2-negative, LA unresectable or metastatic gastric or GEJ adenocarcinoma [18, 19]. In SPOTLIGHT, treatment with zolbetuximab plus mFOLFOX6 (modified folinic acid [or levofolinate], fluorouracil, and oxaliplatin) versus placebo plus mFOLFOX6 significantly improved median progression-free survival (PFS; 10.6 versus 8.7 months; hazard ratio [HR], 0.75; 95% confidence interval [CI] 0.60–0.94; *P* = 0.0066) and median overall survival (OS; 18.2 versus 15.5 months; HR, 0.75; 95% CI 0.60–0.94; *P* = 0.0053) [18]. The most common grade  ≥ 3 treatment-emergent adverse events in SPOTLIGHT were neutropenia, decreased neutrophil count, nausea, and vomiting [18]. In GLOW, treatment with zolbetuximab plus CAPOX (capecitabine and oxaliplatin) versus placebo plus CAPOX significantly improved median PFS (8.2 versus 6.8 months; HR, 0.69; 95% CI 0.54–0.87; *P* = 0.0007) and median OS (14.4 versus 12.2 months; HR, 0.77; 95% CI 0.62–0.97; *P* = 0.0118) [19]. The most common grade  ≥3 treatment-emergent adverse events with zolbetuximab plus CAPOX in GLOW were vomiting, anemia, decreased neutrophil count, and nausea [19].

Consistent with individual analyses, a combined analysis of the 1072 patients in SPOTLIGHT and GLOW showed a median PFS of 9.2 months with zolbetuximab plus chemotherapy and 8.2 months with placebo plus chemotherapy (HR, 0.71; 95% CI 0.61–0.83) [20]. The median OS was 16.4 months with zolbetuximab plus chemotherapy and 13.7 months with placebo plus chemotherapy (HR, 0.77; 95% CI 0.67–0.89) [20]. In the combined analysis, the most common adverse events with zolbetuximab plus chemotherapy were nausea and vomiting [20].

Together, the phase 3 SPOTLIGHT and GLOW studies have demonstrated the benefit of zolbetuximab plus chemotherapy in prolonging PFS and OS; however, the individual studies were limited in their ability to offer conclusions regarding the effectiveness of zolbetuximab in small subgroups, including regional subgroups [18, 19]. Here we report the efficacy and safety of zolbetuximab plus chemotherapy compared with placebo plus chemotherapy as first-line treatment for CLDN18.2-positive, HER2-negative, LA unresectable or metastatic gastric or GEJ adenocarcinoma in Japanese patients in the SPOTLIGHT and GLOW trials.

## Methods

### Study design

The study designs for the global, randomized, double-blind, phase 3 SPOTLIGHT and GLOW trials have been published previously [18, 19]. In brief, eligible patients were aged  ≥18 years (or those considered an adult according to local regulations) and had CLDN18.2-positive, HER2-negative, previously untreated, LA unresectable or metastatic gastric or GEJ adenocarcinoma [18, 19]. CLDN18.2 positivity was defined as  ≥75% of tumor cells having moderate to strong membranous CLDN18 staining tested centrally by immunohistochemistry using the VENTANA^®^ CLDN18 (43-14A) RxDx Assay (Roche Diagnostics, Indianapolis, IN, USA).

### Procedures

Patients in the SPOTLIGHT trial received an intravenous infusion of zolbetuximab (800 mg/m^2^ on cycle 1 day 1 followed by 600 mg/m^2^ on cycle 1 day 22 and days 1 and 22 of subsequent cycles) plus an intravenous infusion of mFOLFOX6 on days 1, 15, and 29 (folinic acid 400 mg/m^2^ or levofolinate 200 mg/m^2^; fluorouracil 400 mg/m^2^ bolus followed by 2400 mg/m^2^ in an infusion over approximately 48 h; and oxaliplatin 85 mg/m^2^) for four 42-day cycles or placebo plus mFOLFOX6 [18]. Patients in the GLOW trial received an intravenous infusion of zolbetuximab 800 mg/m^2^ (cycle 1 day 1) followed by 600 mg/m^2^ (day 1 of subsequent cycles) plus CAPOX (oral capecitabine 1000 mg/m^2^ twice daily on days 1 to 14 of each cycle; intravenous infusion of oxaliplatin 130 mg/m^2^ on day 1 of each cycle) for eight 21-day cycles or placebo plus CAPOX [19].

In the SPOTLIGHT and GLOW trials, tumor response was assessed by imaging at screening and then every 9 weeks for the first 54 weeks. Thereafter, tumor response was assessed every 12 weeks until disease progression or the start of a new anticancer therapy [18, 19].

### Outcomes

The primary endpoint in both SPOTLIGHT and GLOW was PFS per Response Evaluation Criteria in Solid Tumours (RECIST) version 1.1 as determined by an independent review committee (IRC). As a sensitivity analysis, PFS was also determined by investigator assessment. A key secondary endpoint was OS; additional secondary endpoints included objective response rate and duration of response. Objective response and duration of response were also assessed by RECIST version 1.1 as determined by an IRC.

Adverse events were graded according to the National Cancer Institute Common Terminology Criteria for Adverse Events version 4.03. The Medical Dictionary for Regulatory Activities terminology version 25.0 was used to define adverse event preferred terms.

### Statistical analysis

This combined subgroup analysis of the SPOTLIGHT and GLOW trials was prespecified in the statistical analysis plan (SAP). The SAP specified that OS, PFS, objective response rate, and duration of response be evaluated in Japanese and non-Japanese patient subgroups. All other analyses are post hoc analyses conducted for exploratory purposes. These subgroup analyses were not powered for formal hypothesis testing; they should be considered exploratory and descriptive in nature.

The Kaplan–Meier method was used to estimate median PFS, OS, and duration of response; HRs with 95% CIs were based on a stratified Cox proportional hazards model, stratified by number of metastatic sites, prior gastrectomy, and study (SPOTLIGHT or GLOW) for the combined subgroup analyses. The Clopper-Pearson method was used to assess 95% CIs for the objective response rate and disease control rate.

PFS, OS, objective response rate, and duration of response were assessed in the full analysis set, which was composed of all randomized patients. Additionally, objective response rate and duration of response were assessed in the subset of patients with a measurable lesion. Safety was assessed in the safety analysis set, which was composed of patients who received at least one dose of any study drug.

Statistical data analyses were performed with SAS version 9.3 or later (SAS Institute, Cary, NC, USA).

## Results

### Patients and treatment

A total of 1072 patients were enrolled in the SPOTLIGHT and GLOW trials, 537 in the zolbetuximab group and 535 in the placebo group. This combined subgroup analysis includes 116 Japanese patients in the full analysis set who were enrolled in SPOTLIGHT (n = 65; data cutoff: September 8, 2023) or GLOW (n = 51; data cutoff: January 12, 2024), with 114 of these patients in the safety analysis set. Baseline characteristics were generally similar between the Japanese subgroups of the SPOTLIGHT and GLOW trials (Table 1). The baseline characteristics of the Japanese subgroups were also similar to those of the overall SPOTLIGHT and GLOW populations, although the Japanese subgroup had a higher median age, a higher proportion with an Eastern Cooperative Oncology Group performance status of 0, and a higher proportion with diffuse tumor type [18, 19].

**Table 1:** Baseline characteristics

Characteristic		SPOTLIGHT		GLOW	
		Zolbetuximab + mFOLFOX6 (n = 32)	Placebo + mFOLFOX6 (n = 33)	Zolbetuximab + CAPOX (n = 24)	Placebo + CAPOX (n = 27)
Age, years, median (range)		67.5 (33–80)	68.0 (38–86)	65.0 (37–81)	65.0 (33–79)
Sex, n (%)	Male	26 (81.3)	20 (60.6)	15 (62.5)	15 (55.6)
Organs with metastases, n (%)	0–2	30 (93.8)	28 (84.8)	22 (91.7)	22 (81.5)
	≥ 3	2 (6.3)	5 (15.2)	2 (8.3)	5 (18.5)
Prior gastrectomy, n (%)	Yes	10 (31.3)	8 (24.2)	8 (33.3)	8 (29.6)
Primary site, n (%)	Stomach	27 (84.4)	30 (90.9)	22 (91.7)	25 (92.6)
	GEJ	5 (15.6)	3 (9.1)	2 (8.3)	2 (7.4)
Lauren classification, n (%)	Diffuse	22 (68.8)	20 (60.6)	15 (62.5)	18 (66.7)
	Intestinal	7 (21.9)	11 (33.3)	7 (29.2)	7 (25.9)
	Mixed/other	3 (9.4)	2 (6.1)	2 (8.3)	2 (7.4)
ECOG PS, n (%)^a^	0	21 (67.7)	20 (62.5)	17 (70.8)	21 (77.8)
	1	10 (32.3)	12 (37.5)	7 (29.2)	6 (22.2)

^a^Data are missing for one patient in each arm of the SPOTLIGHT trial

*CAPOX* capecitabine and oxaliplatin, *ECOG PS* Eastern Cooperative Oncology Group performance status, *GEJ* gastroesophageal junction, *mFOLFOX6* modified folinic acid (or levofolinate), fluorouracil, and oxaliplatin

### Progression-free survival

**Fig. 1:**
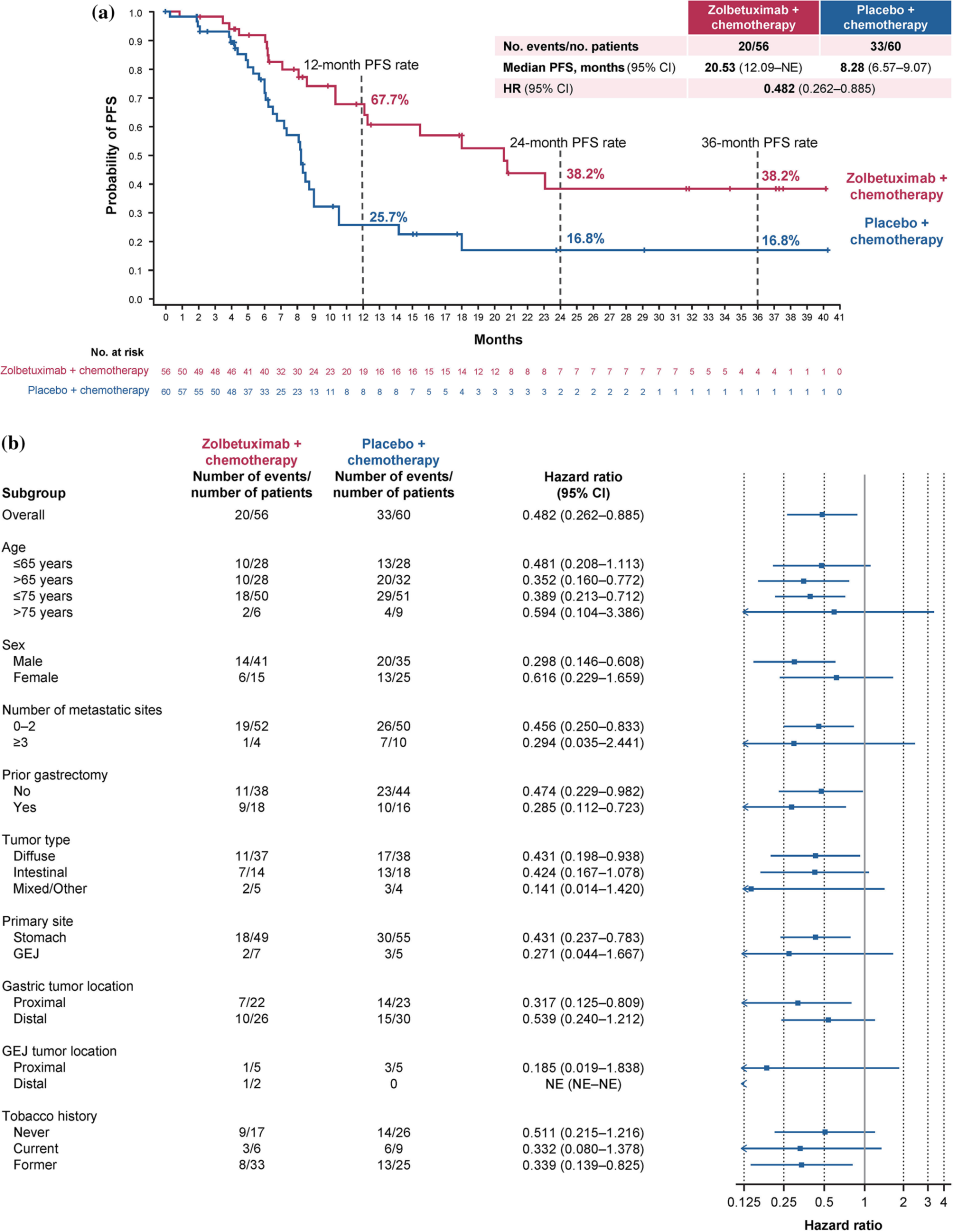
PFS by IRC in the combined Japanese subgroup. **a** Kaplan–Meier plot of PFS by treatment group. **b** Subgroup analyses of PFS by treatment group. *CI* confidence interval, *GEJ* gastroesophageal junction, *HR* hazard ratio, *IRC* independent review committee, *NE* not estimable, *PFS* progression-free survival

In the combined Japanese subgroup, the median follow-up time for PFS was 12.52 months (95% CI 8.15–20.86) in the zolbetuximab group and 15.05 months (95% CI 8.11–23.75) in the placebo group. PFS by independent review committee was improved in the zolbetuximab group versus the placebo group (Fig. 1a). The median PFS was 20.53 months (95% CI 12.09–not estimable [NE]) in the zolbetuximab group versus 8.28 months (95% CI 6.57–9.07) in the placebo group (HR, 0.482; 95% CI 0.262–0.885). The estimated 12-month PFS was 67.7% (95% CI 50.3–80.1%) in the zolbetuximab group versus 25.7% (95% CI 12.9–40.5%) in the placebo group. The estimated 24- and 36-month PFS rates were 38.2% (95% CI 20.0–56.3%; for both time points) in the zolbetuximab group versus 16.8% (95% CI 5.8–32.8%; for both time points) in the placebo group. The PFS benefit of zolbetuximab plus chemotherapy versus placebo plus chemotherapy was generally consistent across subgroups defined by age and other patient characteristics (Fig. 1b).

PFS based on investigator assessment was also improved in the zolbetuximab group versus the placebo group, and PFS by independent review committee was generally consistent between individual studies (Fig. S1).

### Overall survival

**Fig. 2:**
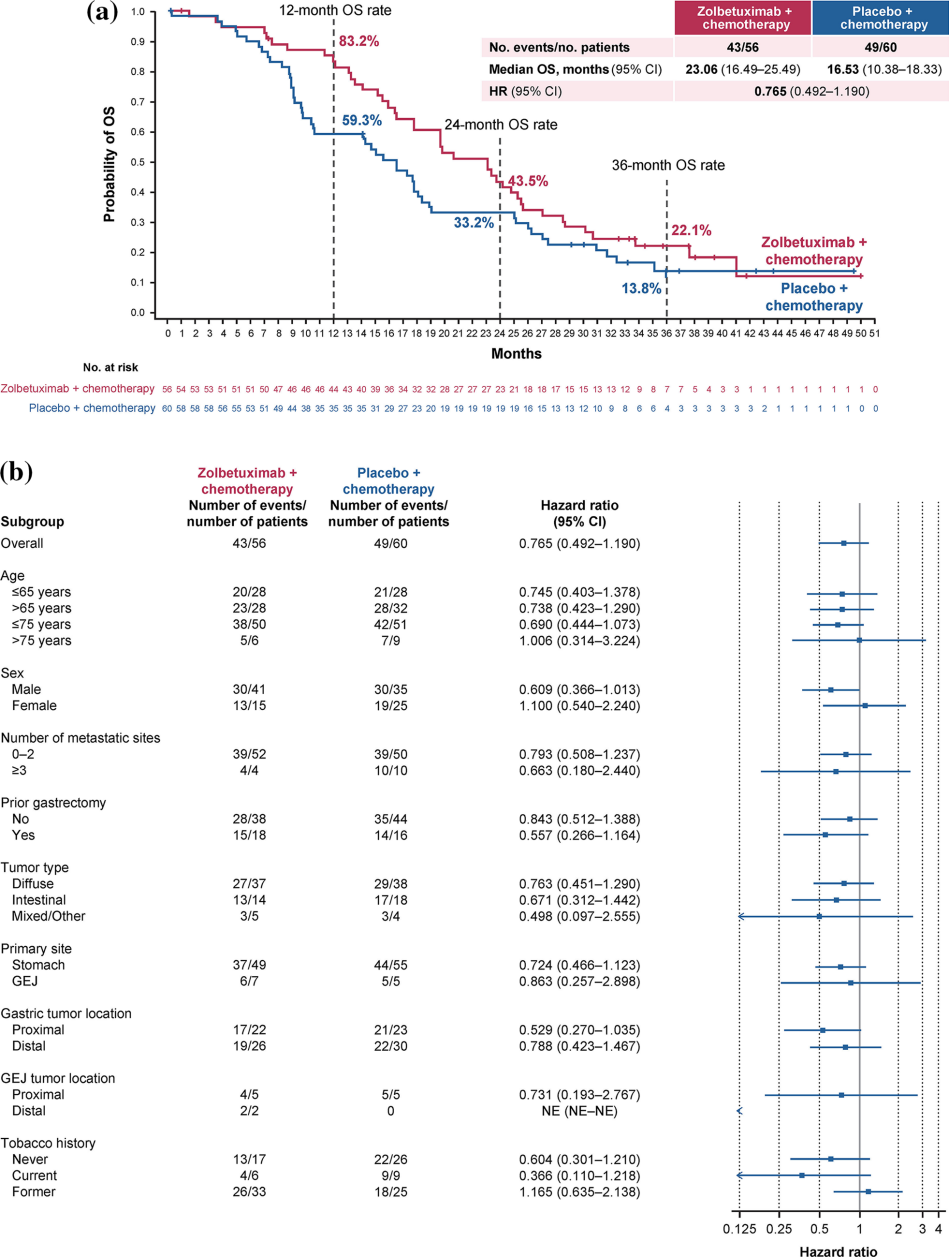
OS in the combined Japanese subgroup. **a** Kaplan–Meier plot of OS by treatment group. **b** Subgroup analyses of OS by treatment group. *CI* confidence interval, *GEJ* gastroesophageal junction, *HR* hazard ratio, *NE* not estimable, *OS* overall survival

The median follow-up time for OS was 37.59 months (95% CI 33.71–41.76) in the zolbetuximab group and 35.94 months (95% CI 33.18–43.70) in the placebo group in this combined Japanese subgroup analysis. The median OS was 23.06 months (95% CI 16.49–25.49) in the zolbetuximab group versus 16.53 months (95% CI 10.38–18.33) in the placebo group (HR, 0.765; 95% CI 0.492–1.190; Fig. 2a). The estimated 12-month OS was 83.2% (95% CI 70.2–90.9%) in the zolbetuximab group versus 59.3% (95% CI 45.7–70.6%) in the placebo group. The estimated 24- and 36-month OS rates were 43.5% (95% CI 30.0–56.2%) and 22.1% (95% CI 12.0–34.2%), respectively, in the zolbetuximab group versus 33.2% (95% CI 21.5–45.3%) and 13.8% (95% CI 6.0–24.8%), respectively, in the placebo group. OS outcomes were generally consistent across subgroups defined by age and other patient characteristics and between individual studies (Fig. 2b and Fig. S2).

### Response rates

In patients with measurable disease in the combined Japanese subgroup, the objective response rate (based on complete or partial response) was 70.0% (95% CI 53.5–83.4%) in the zolbetuximab group versus 57.4% (95% CI 42.2–71.7%) in the placebo group (Table 2). Complete response was achieved in 12.5% of patients in the zolbetuximab group versus 0% of patients in the placebo group. A waterfall plot of the best percent change from baseline in tumor size is shown in Fig. S3. The median duration of response was 10.3 months (95% CI 6.1–NE) in the zolbetuximab group versus 6.1 months (95% CI 3.9–8.6) in the placebo group (HR, 0.639; 95% CI 0.265–1.543). The disease control rate was similar between groups: 90.0% (95% CI 76.3–97.2%) in the zolbetuximab group and 87.2% (95% CI 74.3–95.2%) in the placebo group. Response rates in the full analysis set are summarized in Table S1.

**Table 2:** Response rates and duration of response by IRC^a^

Response	Zolbetuximab+chemotherapy^b^ (n = 40)	Placebo+chemotherapy^b^ (n = 47)
ORR,^c,d^ n	28	27
% (95% CI)	70.0 (53.5–83.4)	57.4 (42.2–71.7)
DCR,^c,e^ n	36	41
% (95% CI)	90.0 (76.3–97.2)	87.2 (74.3–95.2)
BOR,^c,f^ n (%)		
CR	5 (12.5)	0
PR	23 (57.5)	27 (57.4)
SD	8 (20.0)	14 (29.8)
PD	1 (2.5)	3 (6.4)
Not evaluable	0	1 (2.1)
mDOR (95% CI), months	10.3 (6.1–NE)	6.1 (3.9–8.6)

^a^Among patients with measurable disease

^b^Chemotherapy was either mFOLFOX6 (SPOTLIGHT) or CAPOX (GLOW)

^c^Per RECIST version 1.1

^d^Defined as the percentage of patients with BOR of CR or PR

^e^Defined as the percentage of patients with BOR of CR, PR, or SD (≥ 8 weeks for SD)

^f^Data were not available (no postbaseline imaging assessment) for three patients in the zolbetuximab group and two patients in the placebo group

*BOR* best overall response, *CAPOX* capecitabine and oxaliplatin, *CI* confidence interval, *CR* complete response, *DCR* disease control rate, *IRC* independent review committee, *mDOR* median duration of response, *mFOLFOX6* modified folinic acid (or levofolinate), fluorouracil, and oxaliplatin, *NE* not estimable, *ORR* objective response rate, *PD* progressive disease, *PR* partial response, *RECIST* Response Evaluation Criteria in Solid Tumours, *SD* stable disease

### Subsequent anticancer therapy

In the combined Japanese subgroup, subsequent anticancer therapy was used in 76.8% of patients in the zolbetuximab group and 83.3% of patients in the placebo group (Table S2). Among the combined non-Japanese patient subgroup of the SPOTLIGHT and GLOW trials, subsequent anticancer therapy was used in 50.1% of patients in the zolbetuximab group and 56.2% of patients in the placebo group. The most common subsequent systemic therapies used in the combined Japanese subgroup included paclitaxel, nivolumab, and ramucirumab.

### Exposure and safety

In the safety analysis set of the combined Japanese subgroup, the median duration of treatment exposure was 218 days for zolbetuximab and 156 days for placebo, with 98.2% of patients in the zolbetuximab group and 100% of patients in the placebo group receiving a relative dose intensity  >80% of zolbetuximab or placebo, respectively (Table S3). In the non-Japanese population, the median duration of zolbetuximab or placebo exposure was 169 days in the zolbetuximab group and 176 days in the placebo group, with 88.9% of patients in the zolbetuximab group and 99.1% of patients in the placebo group receiving a relative dose intensity  >80% of zolbetuximab or placebo, respectively. Median cumulative actual dose of zolbetuximab was numerically higher in the Japanese subgroup versus the non-Japanese subgroup (6652 versus 5000 mg/m^2^). In the Japanese subgroup, 7.3% of patients prematurely discontinued a zolbetuximab infusion in the zolbetuximab group versus 0% who discontinued a placebo infusion in the placebo group; interruption of infusion occurred in 47.3% and 1.7% of patients, respectively. In the non-Japanese subgroup, 15.1% of patients prematurely discontinued an infusion in the zolbetuximab group versus 0.6% in the placebo group; interruption of infusion occurred in 52.1% and 5.6% of patients, respectively.

**Fig. 3:**
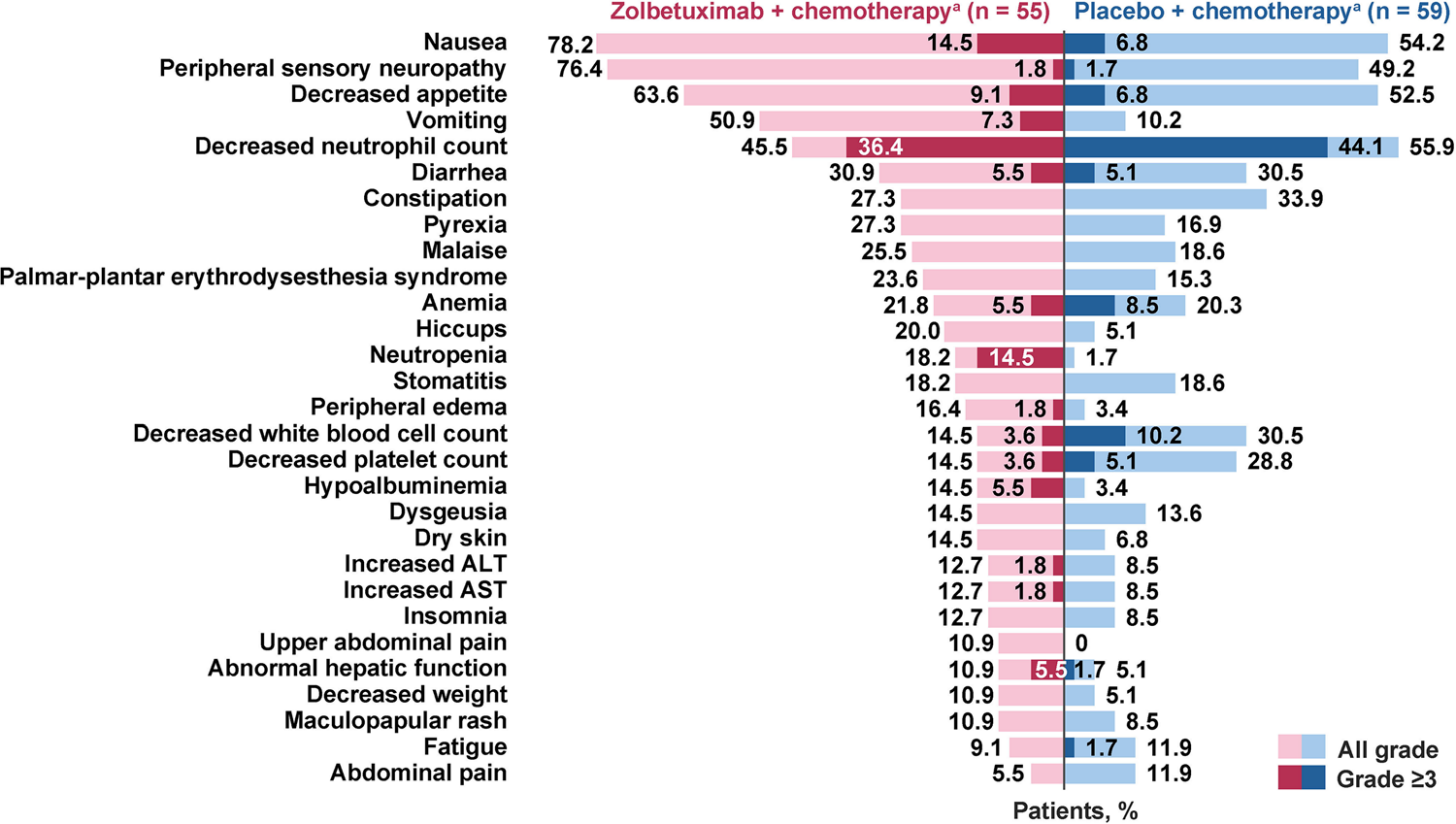
Adverse events in  >10% of patients in either treatment group in the combined Japanese subgroup safety analysis set. ^a^Chemotherapy was either mFOLFOX6 (SPOTLIGHT) or CAPOX (GLOW). *ALT* alanine aminotransferase, *AST* aspartate aminotransferase, *CAPOX* capecitabine and oxaliplatin, *mFOLFOX6* modified folinic acid (or levofolinate), fluorouracil, and oxaliplatin

Treatment-emergent adverse events were reported in 100% (55/55) of patients in the zolbetuximab group and 96.6% (57/59) of patients in the placebo group. The treatment-emergent adverse events (any grade) with the greatest difference in frequency between the zolbetuximab group and the placebo group (>20 percentage points) were vomiting, peripheral sensory neuropathy, and nausea. Grade  ≥3 adverse events were reported in 69.1% (38/55) of patients in the zolbetuximab group and 66.1% (39/59) of patients in the placebo group; serious adverse events were reported in 32.7% (18/55) and 28.8% (17/59) of patients, respectively. The most common grade  ≥3 treatment-emergent adverse events with zolbetuximab plus chemotherapy (reported in  >10% of patients) were decreased neutrophil count, nausea, and neutropenia (Fig. 3). The grade  ≥3 treatment-emergent adverse events with the greatest difference in frequency between the zolbetuximab group and the placebo group (>5 percentage points for zolbetuximab versus placebo) were neutropenia, nausea, vomiting, and hypoalbuminemia.

Prophylactic antiemetic medication was used in all 55 patients in the safety analysis set who were treated with zolbetuximab on the initial infusion day (Table S4). Compared with non-Japanese patients, a greater percentage of Japanese patients received prophylactic neurokinin-1 (NK-1) receptor blockers (70.9% versus 59.7%), systemic corticosteroids (50.9% versus 29.3%), and systemic antihistamines (34.5% versus 17.5%).

Treatment-emergent adverse events leading to discontinuation of any study drug at any time on study occurred in 32.7% (18/55) of patients in the zolbetuximab group and 30.5% (18/59) of patients in the placebo group. Of note, few treatment-emergent adverse events led to early treatment discontinuation (within 9 weeks) of zolbetuximab or placebo in either treatment subgroup (Table S5). In the zolbetuximab group, one treatment-related death occurred in a patient who received zolbetuximab + mFOLFOX6 and experienced acute myocardial infarction, disseminated intravascular coagulation, and pneumonia.

## Discussion

Zolbetuximab plus chemotherapy demonstrated an improvement in PFS and a numerical improvement in OS in Japanese patients with CLDN18.2-positive, HER2-negative, previously untreated, LA unresectable or metastatic gastric or GEJ adenocarcinoma in this combined Japanese subgroup analysis of the phase 3 SPOTLIGHT and GLOW trials. Additionally, no new safety signals were identified in Japanese patients.

Among Japanese patients, the reduction in the risk of disease progression or death was  ~52% with the addition of zolbetuximab to chemotherapy, which was consistent with the trend of reduction in the risk of progression or death in the overall population in the SPOTLIGHT and GLOW trials (~25% and  ~31%, respectively) [18, 19]. The reduction in the risk of death among Japanese patients (~24%) was similar to that of the overall population in the SPOTLIGHT and GLOW trials (~25% and  ~23%, respectively) [18, 19]. A review by Kubota et al. discussed that in recent phase 3 trials, the survival benefit of the experimental arm in the Japanese population was not necessarily consistent with that of the overall population, although these were subgroup analyses of a limited number of patients [21]. The authors mentioned that this result may be due to differences in post-study treatment in Japan [21]. Therefore, we believe that the consistency of the survival benefit in the Japanese population with that of the overall population in the SPOTLIGHT and GLOW trials may be an important finding from this analysis. Further, Japanese patients treated with zolbetuximab plus chemotherapy tended to experience deeper responses (as evidenced by a larger percent change from baseline in tumor size) than those treated with placebo plus chemotherapy (Fig. S3). More broadly, response rates with zolbetuximab plus chemotherapy have tended to be similar to or slightly higher than those with placebo plus chemotherapy [18, 19]. Objective responses may have contributed to the reduced risk of disease progression or death and a numerical improvement in OS in Japanese patients who received zolbetuximab plus chemotherapy.

The zolbetuximab treatment group experienced a higher rate of infusion interruption than the placebo group in both the Japanese and the non-Japanese populations. The infusion interruptions may have been necessary to manage nausea and vomiting, which are typical adverse events reported following treatment with zolbetuximab. However, the Japanese patient population also had a longer zolbetuximab drug exposure period and a higher cumulative actual zolbetuximab dose compared with the non-Japanese population. Further, the proportion of patients with a relative dose intensity  >80% was numerically higher in the Japanese population compared with the non-Japanese population. Adequate and sustained exposure to zolbetuximab may be important for achieving a better survival benefit.

Adverse events in Japanese patients were generally consistent with those in the overall populations of SPOTLIGHT and GLOW [18, 19]. The most frequent adverse events in Japanese patients who received zolbetuximab plus chemotherapy were nausea, peripheral sensory neuropathy, decreased appetite, and vomiting. The only adverse events with a difference in frequency of  >20 percentage points between the zolbetuximab group and the placebo group were vomiting, peripheral sensory neuropathy, and nausea.

Given the frequency of nausea and vomiting, prophylactic antiemetic medication was used on the initial infusion day in all Japanese patients treated with zolbetuximab. In the Japanese population, a higher proportion of patients received prophylactic corticosteroids (50.9% versus 29.3%) and antihistamines (34.5% versus 17.5%) compared with the non-Japanese population. Further, no early permanent discontinuations of zolbetuximab or placebo due to nausea or vomiting were observed in the Japanese population. Therefore, the prophylactic use of appropriate antiemetics, including corticosteroids, may prevent early discontinuation and enable longer-term treatment with zolbetuximab. This is consistent with findings from a prior analysis of nausea and vomiting in the SPOTLIGHT and GLOW overall population, which found that a slower infusion rate and the use of antiemetic combination therapies (eg, a serotonin antagonist plus a steroid, and a serotonin antagonist plus an NK-1 receptor blocker plus a steroid) may help mitigate nausea and vomiting with zolbetuximab treatment [22]. Recently, the Japan Society of Clinical Oncology’s Committee of Clinical Practice Guidelines for Antiemesis published a flash report on the management of nausea and vomiting during zolbetuximab treatment. This report highlighted the importance of infusion rate and antiemetics for the management of nausea and vomiting during the first cycle of zolbetuximab plus chemotherapy regimens [23]. We believe that our current analysis further supports the recommendations in this flash report.

The use of new/subsequent anticancer therapy was higher among Japanese patients versus non-Japanese patients treated with zolbetuximab (76.8% versus 50.1%) in the SPOTLIGHT and GLOW trials. Compared with non-Japanese patients, Japanese patients were much more likely to receive ramucirumab, paclitaxel, and nivolumab as subsequent anticancer therapies. The Japanese Gastric Cancer Treatment Guidelines recommend ramucirumab plus paclitaxel as second-line therapy and nivolumab as one of three options for third-line or later therapy for unresectable advanced/recurrent gastric cancer (other options are chemotherapy) [24]. The higher use of ramucirumab, paclitaxel, and nivolumab among Japanese patients likely reflects the use of Japanese guideline-recommended therapies for gastric cancer. In recent years, several targeted therapies have been approved for first-line treatment of gastric cancer, including nivolumab, pembrolizumab, trastuzumab, and zolbetuximab [9, 25]. With the expansion of first-line treatment options, it will be important to consider the optimal treatment sequence to achieve the best outcomes for patients with gastric cancer [26].

Limitations of this study include the small size of the Japanese subgroup. Because this analysis was exploratory in nature, the results should be interpreted as hypothesis generating rather than confirmatory. The combined Japanese subgroup analysis should be interpreted in the context of the overall SPOTLIGHT and GLOW data.

In conclusion, zolbetuximab plus chemotherapy improved PFS versus placebo plus chemotherapy and showed a numerical improvement in OS in Japanese patients. These results support zolbetuximab plus chemotherapy as a potential new standard of care in the first-line setting for Japanese patients with CLDN18.2-positive, HER2-negative, LA unresectable or metastatic gastric or GEJ adenocarcinoma.

## Electronic Supplementary Material

Below is the link to the electronic supplementary material.

Supplementary Material 1

## Data Availability

Details for how researchers may request access to anonymized participant level data, trial level data and protocols from Astellas sponsored clinical trials can be found at https://www.clinicaltrials.astellas.com/transparency/.

## References

[1] Bray F, Laversanne M, Sung H, Ferlay J, Siegel RL, Soerjomataram I, et al. Global cancer statistics 2022: GLOBOCAN estimates of incidence and mortality worldwide for 36 cancers in 185 countries. CA Cancer J Clin. 2024;74:229–63. 38572751 10.3322/caac.21834

[2] Xia JY, Aadam AA. Advances in screening and detection of gastric cancer. J Surg Oncol. 2022;125:1104–9. 35481909 10.1002/jso.26844 PMC9322671

[3] Mabe K, Inoue K, Kamada T, Kato K, Kato M, Haruma K. Endoscopic screening for gastric cancer in Japan: current status and future perspectives. Dig Endosc. 2022;34:412–9. 34143908 10.1111/den.14063

[4] Bang YJ, Van Cutsem E, Feyereislova A, Chung HC, Shen L, Sawaki A, et al. Trastuzumab in combination with chemotherapy versus chemotherapy alone for treatment of HER2-positive advanced gastric or gastro-oesophageal junction cancer (ToGA): a phase 3, open-label, randomised controlled trial. Lancet. 2010;376:687–97. 20728210 10.1016/S0140-6736(10)61121-X

[5] Janjigian YY, Shitara K, Moehler M, Garrido M, Salman P, Shen L, et al. First-line nivolumab plus chemotherapy versus chemotherapy alone for advanced gastric, gastro-oesophageal junction, and oesophageal adenocarcinoma (CheckMate 649): a randomised, open-label, phase 3 trial. Lancet. 2021;398:27–40. 34102137 10.1016/S0140-6736(21)00797-2 PMC8436782

[6] Zhang J, Wang G, Xie X, Pan W, Dong Q, Zhang N, et al. Treatment patterns and outcomes in advanced or metastatic gastric/gastroesophageal junction adenocarcinoma in China. Future Oncol. 2025;21:1179–88. 40091795 10.1080/14796694.2025.2476930 PMC11988209

[7] Luna J, Picker N, Wilke T, Lutz M, Hess J, Mörtl B, et al. Real-world evidence of treatment patterns and survival of metastatic gastric cancer patients in Germany. BMC Cancer. 2024;24:462. 38614966 10.1186/s12885-024-12204-x PMC11016202

[8] Astellas. Astellas’ VYLOY™ (zolbetuximab) approved in Japan for treatment of gastric cancer [press release]. 2024. https://www.astellas.com/en/news/29026. Accessed 25 Jul 2025.

[9] Vyloy [package insert]. Northbrook, IL: Astellas Pharma, Inc.; 2025.

[10] Vyloy [summary of product characteristics]. Leiden, Netherlands: Astellas Pharma Europe; 2025.

[11] Sahin U, Koslowski M, Dhaene K, Usener D, Brandenburg G, Seitz G, et al. Claudin-18 splice variant 2 is a pan-cancer target suitable for therapeutic antibody development. Clin Cancer Res. 2008;14:7624–34. 19047087 10.1158/1078-0432.CCR-08-1547

[12] Sahin U, Türeci Ö, Manikhas G, Lordick F, Rusyn A, Vynnychenko I, et al. FAST: a randomised phase II study of zolbetuximab (IMAB362) plus EOX versus EOX alone for first-line treatment of advanced CLDN18.2-positive gastric and gastro-oesophageal adenocarcinoma. Ann Oncol. 2021;32:609–19. 33610734 10.1016/j.annonc.2021.02.005

[13] Shitara K, Xu RH, Ajani JA, Moran D, Guerrero A, Li R, et al. Global prevalence of claudin 18 isoform 2 in tumors of patients with locally advanced unresectable or metastatic gastric or gastroesophageal junction adenocarcinoma. Gastric Cancer. 2024;27:1058–68. 38954176 10.1007/s10120-024-01518-1 PMC11335819

[14] Shitara K, Kawazoe A, Hirakawa A, Nakanishi Y, Furuki S, Fukuda M, et al. Phase 1 trial of zolbetuximab in Japanese patients with CLDN18.2+ gastric or gastroesophageal junction adenocarcinoma. Cancer Sci. 2023;114:1606–15. 36478334 10.1111/cas.15684 PMC10067400

[15] Sahin U, Schuler M, Richly H, Bauer S, Krilova A, Dechow T, et al. A phase I dose-escalation study of IMAB362 (Zolbetuximab) in patients with advanced gastric and gastro-oesophageal junction cancer. Eur J Cancer. 2018;100:17–26. 29936063 10.1016/j.ejca.2018.05.007

[16] Mitnacht-Kraus R, Kreuzberg M, Utsch M, Sahin U, Türeci Ö. Preclinical characterization of IMAB362 for the treatment of gastric carcinoma [abstract]. Ann Oncol. 2017;28(suppl 5):v126.

[17] National Medical Products Administration. Zolbetuximab for injection approved for marketing by China NMPA. 2025. https://english.nmpa.gov.cn/2025-06/11/c_1101548.htm. Accessed 26 Nov 2025.

[18] Shitara K, Lordick F, Bang YJ, Enzinger P, Ilson D, Shah MA, et al. Zolbetuximab plus mFOLFOX6 in patients with CLDN18.2-positive, HER2-negative, untreated, locally advanced unresectable or metastatic gastric or gastro-oesophageal junction adenocarcinoma (SPOTLIGHT): a multicentre, randomised, double-blind, phase 3 trial. Lancet. 2023;401:1655–68. 37068504 10.1016/S0140-6736(23)00620-7

[19] Shah MA, Shitara K, Ajani JA, Bang YJ, Enzinger P, Ilson D, et al. Zolbetuximab plus CAPOX in CLDN18.2-positive gastric or gastroesophageal junction adenocarcinoma: the randomized, phase 3 GLOW trial. Nat Med. 2023;29:2133–41. 37524953 10.1038/s41591-023-02465-7 PMC10427418

[20] Shitara K, Shah MA, Lordick F, Van Cutsem E, Ilson DH, Klempner SJ, et al. Zolbetuximab in gastric or gastroesophageal junction adenocarcinoma. N Engl J Med. 2024;391:1159–62. 39282934 10.1056/NEJMc2409512 PMC11937907

[21] Kubota Y, Aoki Y, Kawazoe A, Shitara K. Role of nivolumab in the management of first-line unresectable advanced or recurrent gastric cancer in combination with chemotherapy: lessons from the Japanese experience. Cancer Manag Res. 2022;14:3083–94. 36275782 10.2147/CMAR.S351791 PMC9584771

[22] Shitara K, Pophale R, Matsangou M, Park JW, Oh M, Bhattacharya PP, et al. Management of nausea and vomiting (N/V) following first-line (1L) zolbetuximab+chemotherapy treatment in claudin-18.2 (CLDN18.2)+, HER2−, locally advanced (LA) unresectable or metastatic gastric or gastroesophageal junction (mG/GEJ) adenocarcinoma: analysis from the phase 3 SPOTLIGHT and GLOW studies. J Clin Oncol. 2024;42(3suppl):372.

[23] Boku N, Aogi K, Japan Society of Clinical Oncology. Anti-emetic therapy during first-line chemotherapy containing zolbetuximab for HER2-negative, Claudin 18.2-positive unresectable advanced/recurrent gastric cancer. Flash report of the clinical practice guidelines for antiemesis. 2025. https://www.jsco.or.jp/Portals/0/5_Guideline/Flash%20report%20of%20the%20CPG%20for%20antiemesis_2025Feb%20(002).pdf. Accessed 13 Feb 2026.

[24] Japanese Gastric Cancer Association. Japanese Gastric Cancer Treatment Guidelines 2025 (7th edition). Gastric Cancer. 2026;29:271–99. 10.1007/s10120-025-01698-4 PMC12956939 41569370

[25] Högner A, Moehler M. Immunotherapy in gastric cancer. Curr Oncol. 2022;29:1559–74. 35323331 10.3390/curroncol29030131 PMC8946975

[26] Yoshikawa T, Kikko Y, Makino R, Kimijima Y, Nishiyama E, Matsuda Y, et al. Adjuvant and post-recurrent treatment patterns in patients with resectable gastric cancer in Japan: a retrospective database cohort study. Gastric Cancer. 2024;27:827–39. 38689045 10.1007/s10120-024-01501-w PMC11193688

