# Supplementary material for: Zolbetuximab plus chemotherapy in Japanese patients with claudin 18.2–positive gastric or gastroesophageal junction adenocarcinoma: a combined subgroup analysis of the phase 3 SPOTLIGHT and GLOW trials
Source: Gastric Cancer. 2026 May 7;29(4):777–87. doi: 10.1007/s10120-026-01738-7 (PMC13314679; doi:10.1007/s10120-026-01738-7)

SUPPLEMENTARY MATERIAL

Gastric Cancer

Zolbetuximab plus chemotherapy in Japanese patients with claudin 18.2–positive gastric or gastroesophageal junction adenocarcinoma: a combined subgroup analysis of the phase 3 SPOTLIGHT and GLOW trials

Kensei Yamaguchi, MD[1]; Hirokazu Shoji, MD[2]; Hisateru Yasui, MD[3]; Eiji Oki, MD[4]; Daisuke Sakai, MD[5]; Tetsuya Hamaguchi, MD[6]; Akihito Tsuji, MD[7]; Takashi Oshima, MD[8]; Masahiro Tsuda, MD[9]; Keiko Minashi, MD[10]; Jianning Yang, PhD[11]; Abraham Guerrero, PhD[11]; Yoko Ueno, PhD[12]; Maria Matsangou, MD[11]; Georgia Gourgioti, MS[13]; Yuka Nakanishi, MS, MBA[12]; Satomi Furuki, MS[12]; Kana Kuwamoto, MS, MBA[12]; Shunsuke Yamada, MEng[12]; Kohei Shitara, MD[14]

[1]Cancer Institute Hospital of the Japanese Foundation for Cancer Research, Tokyo, Japan; [2]National Cancer Center Hospital, Tokyo, Japan; [3]Kobe City Medical Center General Hospital, Hyogo, Japan; [4]Kyushu University, Fukuoka, Japan; [5]Osaka University, Osaka, Japan; [6]Saitama Medical University International Medical Center, Saitama, Japan; [7]Kagawa University, Kagawa, Japan; [8]Kanagawa Cancer Center, Kanagawa, Japan; [9]Hyogo Cancer Center, Hyogo, Japan; [10]Chiba Cancer Center, Chiba, Japan; [11]Astellas Pharma Global Development, Inc., Northbrook, IL, USA;

[12]Astellas Pharma, Inc., Tokyo, Japan; [13]Astellas Pharma Europe Ltd., Addlestone, United Kingdom; [14]National Cancer Center Hospital East, Kashiwa, Japan

Corresponding Author:

Kohei Shitara, MD

Director of the Department of Gastrointestinal Oncology

National Cancer Center Hospital East

Kashiwa, Japan

Email: kshitara@east.ncc.go.jp

Table S1. Response rates and duration of response by IRC in the full analysis set of the combined Japanese subgroup[a]

Response	Zolbetuximab + chemotherapy[b] (n = 56)	Placebo + chemotherapy[b] (n = 60)
ORR,[c,d] n	28	27
% (95% CI)	50.0 (36.3–63.7)	45.0 (32.1–58.4)
DCR,[c,e] n	50	53
% (95% CI)	89.3 (78.1–96.0)	88.3 (77.4–95.2)
BOR,[c,f] n (%)		
CR	5 (8.9)	0
PR	23 (41.1)	27 (45.0)
SD	8 (14.3)	14 (23.3)
Non-CR/non-PD	14 (25.0)	12 (20.0)
PD	1 (1.8)	3 (5.0)
Not evaluable	0	1 (1.7)
mDOR (95% CI), months	10.3 (6.1–NE)	6.1 (3.9–8.6)

[a]Results for patients with measurable disease are shown in Table 2
[b]Chemotherapy was either mFOLFOX6 (SPOTLIGHT) or CAPOX (GLOW)
[c]Per RECIST version 1.1
[d]Defined as the percentage of patients with BOR of CR or PR
[e]Defined as the percentage of patients with BOR of CR, PR, SD, or non-CR/non-PD (≥8 weeks for SD in measurable disease and non-CR/non-PD in non-measurable disease)
[f]Data were not available (no postbaseline imaging assessment) for five patients in the zolbetuximab group and three patients in the placebo group

BOR best overall response, *CAPOX* capecitabine and oxaliplatin, *CI* confidence interval, *CR* complete response, *DCR* disease control rate, *IRC* independent review committee, *mDOR* median duration of response, *mFOLFOX6* modified folinic acid (or levofolinate), fluorouracil, and oxaliplatin, *NE* not estimable, *ORR* objective response rate, *PD* progressive disease, *PR* partial response, *RECIST* Response Evaluation Criteria in Solid Tumours, *SD* stable disease

Table S2. New/subsequent anticancer therapy

Therapy, n (%)	Combined Japanese subgroup		Non-Japanese subgroup	
	**Zolbetuximab + chemotherapy[a] (n = 56)**	**Placebo + chemotherapy[a] (n = 60)**	**Zolbetuximab + chemotherapy[a] (n = 481)**	**Placebo + chemotherapy[a] (n = 475)**
Any new/subsequent therapy	43 (76.8)	50 (83.3)	241 (50.1)	267 (56.2)
Radiotherapy	3 (5.4)	2 (3.3)	10 (2.1)	31 (6.5)
Most common subsequent systemic therapies[b]				
Taxanes				
Paclitaxel	27 (48.2)	26 (43.3)	76 (15.8)	91 (19.2)
Nab-paclitaxel	8 (14.3)	9 (15.0)	20 (4.2)	15 (3.2)
PD-1/PD-L1 inhibitors				
Nivolumab	26 (46.4)	21 (35.0)	16 (3.3)	17 (3.6)
VEGF/VEGFR inhibitor–based regimens				
Ramucirumab	24 (42.9)	26 (43.3)	39 (8.1)	47 (9.9)
Ramucirumab + paclitaxel	5 (8.9)	7 (11.7)	33 (6.9)	33 (6.9)
Pyrimidine analogues				
Fluorouracil	3 (5.4)	1 (1.7)	28 (5.8)	32 (6.7)
Gimeracil, oteracil potassium, tegafur	1 (1.8)	4 (6.7)	15 (3.1)	10 (2.1)
Tipiracil hydrochloride, trifluridine	1 (1.8)	4 (6.7)	4 (0.8)	8 (1.7)
Platinum compounds				
Cisplatin	2 (3.6)	0	11 (2.3)	10 (2.1)
Immunosuppressants				
Immunotherapy	2 (3.6)	0	—	—
Topoisomerase 1 inhibitors				
Irinotecan hydrochloride trihydrate	0	2 (3.3)	—	—
Other				
Investigational agents	3 (5.4)	2 (3.3)	2 (0.4)	3 (0.6)
Other agents	2 (3.6)	3 (5.0)	12 (2.5)	8 (1.7)
Calcium levofolinate pentahydrate	2 (3.6)	0	—	—

[a]Chemotherapy was either mFOLFOX6 (SPOTLIGHT) or CAPOX (GLOW)
[b]Administered to ≥2% of patients in either treatment group in the combined Japanese subgroup
CAPOX capecitabine and oxaliplatin, *mFOLFOX6* modified folinic acid (or levofolinate), fluorouracil, and oxaliplatin, *Nab* nanoparticle albumin-bound, *PD-1* programmed cell death protein 1, *PD-L1* programmed cell death ligand 1, *VEGF* vascular endothelial growth factor, *VEGFR* vascular endothelial growth factor receptor

Table S3. Exposure, safety analysis set

Parameter	Combined Japanese subgroup		Non-Japanese subgroup	
	**Zolbetuximab + chemotherapy[a]**	**Placebo + chemotherapy[a]**	**Zolbetuximab + chemotherapy[a]**	**Placebo + chemotherapy[a]**
Zolbetuximab or placebo exposure[b]				
n	55	59	477	468
Mean duration (SD), days	351.0 (347.0)	221.5 (222.3)	257.3 (281.1)	232.9 (215.3)
Median duration (min, max), days	218.0 (1, 1261)	156.0 (1, 1290)	169.0 (1,1603)	176.0 (1, 1395)
Oxaliplatin exposure[b]				
n	55	59	469	468
Mean duration (SD), days	132.7 (58.8)	120.6 (51.7)	120.0 (60.2)	120.8 (56.7)
Median duration (min, max), days	139.0 (1, 239)	127.0 (1, 207)	140.0 (1, 267)	139.5 (1, 253)
Cumulative actual dose of zolbetuximab or placebo				
n	55	59	477	468
Mean (SD), mg/m^2	9749 (9261)	6353 (5570)	7280 (7489)	6767 (5616)
Median (min, max), mg/m^2	6652 (788, 33,200)	5000 (800, 34,390)	5000 (51, 46,400)	5570 (28, 37,400)
Relative dose intensity[c] of zolbetuximab or placebo				
n	55	59	477	468
>80%, n (%)	54 (98.2)	59 (100)	424 (88.9)	464 (99.1)
Interrupted infusion of zolbetuximab or placebo				
n	55	59	478	468
n (%)	26 (47.3)	1 (1.7)	249 (52.1)	26 (5.6)
Prematurely discontinued infusion of zolbetuximab or placebo				
n	55	59	478	468
n (%)	4 (7.3)	0	72 (15.1)	3 (0.6)
Time to first dose modification[d] of zolbetuximab or placebo				
n	33	13	308	142
Mean (SD), days	38.8 (105.8)	140.5 (146.6)	36.6 (81.7)	121.7 (130.5)
Median (min, max), days	1.0 (1, 567)	96.0 (22, 514)	1.0 (1, 890)	90.5 (1, 960)

[a]Chemotherapy was either mFOLFOX6 (SPOTLIGHT) or CAPOX (GLOW)
[b]Duration of exposure was defined as (date of last infusion) − (date of first infusion) + 1
[c]Relative dose intensity was defined as (cumulative actual dose / planned cumulative dose) × 100%
[d]Time to first dose modification was defined as (date of first dose modification) − (date of first infusion) + 1
CAPOX capecitabine and oxaliplatin, *max* maximum, *mFOLFOX6* modified folinic acid (or levofolinate), fluorouracil, and oxaliplatin, *min* minimum, *SD* standard deviation

Table S4. Prophylactic medication for nausea or vomiting on day of initial zolbetuximab infusion and frequency of nausea or vomiting

	**Combined Japanese subgroup**			**Non-Japanese subgroup**		
**Medication**	**Patients with concomitant use, n (%)**	**All grade, n (%)[a]**	**Grade ≥3, n (%)[a]**	**Patients with concomitant use, n (%)[b]**	**All grade, n (%)[a]**	**Grade ≥3, n (%)[a]**
Any antiemetic medication	55 (100.0)	32 (58.2)	4 (7.3)	466 (98.3)	285 (61.2)	43 (9.2)
NK-1 receptor blockers	39 (70.9)	19 (48.7)	2 (5.1)	283 (59.7)	169 (59.7)	22 (7.8)
Serotonin (5-HT3) antagonists	55 (100.0)	32 (58.2)	4 (7.3)	458 (96.6)	282 (61.6)	43 (9.4)
Systemic antihistamines	19 (34.5)	11 (57.9)	0	83 (17.5)	48 (57.8)	9 (10.8)
Systemic corticosteroids	28 (50.9)	15 (53.6)	2 (7.1)	139 (29.3)	75 (54.0)	13 (9.4)
Olanzapine	0	0	0	4 (0.8)	4 (100.0)	0

[a]The denominator is the number of patients with concomitant use

[b]The denominator is the number of patients with data available (n = 474)

5-HT3 5-hydroxytryptamine-3, *NK-1* neurokinin-1

Table S5. TEAEs leading to early[a] permanent discontinuation of zolbetuximab or placebo

	**Combined Japanese subgroup**		**Non-Japanese subgroup**	
	**Zolbetuximab + chemotherapy[b]**	**Placebo + chemotherapy[b]**	**Zolbetuximab + chemotherapy[b]**	**Placebo + chemotherapy[b]**
Patients who discontinued treatment early,[a] n/n (%)	11/55 (20.0)	5/59 (8.5)	121/478 (25.3)	72/468 (15.4)
Patients who discontinued treatment early[a] due to TEAEs, n (%)[c]	1 (9.1)	2 (40.0)	45 (37.2)	21 (29.2)
Abnormal hepatic function	1 (9.1)	0	0	0
Decreased appetite	1 (9.1)	0	1 (0.8)	0
Cerebral hemorrhage	0	1 (20.0)	0	0
Chronic kidney disease	0	1 (20.0)	0	0
Vomiting	0	0	16 (13.2)	2 (2.8)
Nausea	0	0	10 (8.3)	1 (1.4)

[a]Within 9 weeks

[b]Chemotherapy was either mFOLFOX6 (SPOTLIGHT) or CAPOX (GLOW)

[c]The denominator is the number of patients who discontinued treatment early. Individual TEAEs reported in >5% of patients who permanently discontinued treatment early in any group are shown

CAPOX capecitabine and oxaliplatin, *mFOLFOX6* modified folinic acid (or levofolinate), fluorouracil, and oxaliplatin, *TEAE* treatment-emergent adverse event

Fig. S1 PFS by investigator assessment in the combined Japanese subgroup and PFS by IRC assessment for each study.

a) Investigator-assessed PFS in the combined Japanese subgroup. **b**) PFS assessed by IRC in the SPOTLIGHT Japanese subgroup. **c**) PFS assessed by IRC in the GLOW Japanese subgroup. *CAPOX* capecitabine and oxaliplatin, *CI* confidence interval, *HR* hazard ratio, *IRC* independent review committee, *mFOLFOX6* modified folinic acid (or levofolinate), fluorouracil, and oxaliplatin, *NE* not estimable, *PFS* progression-free survival

a.

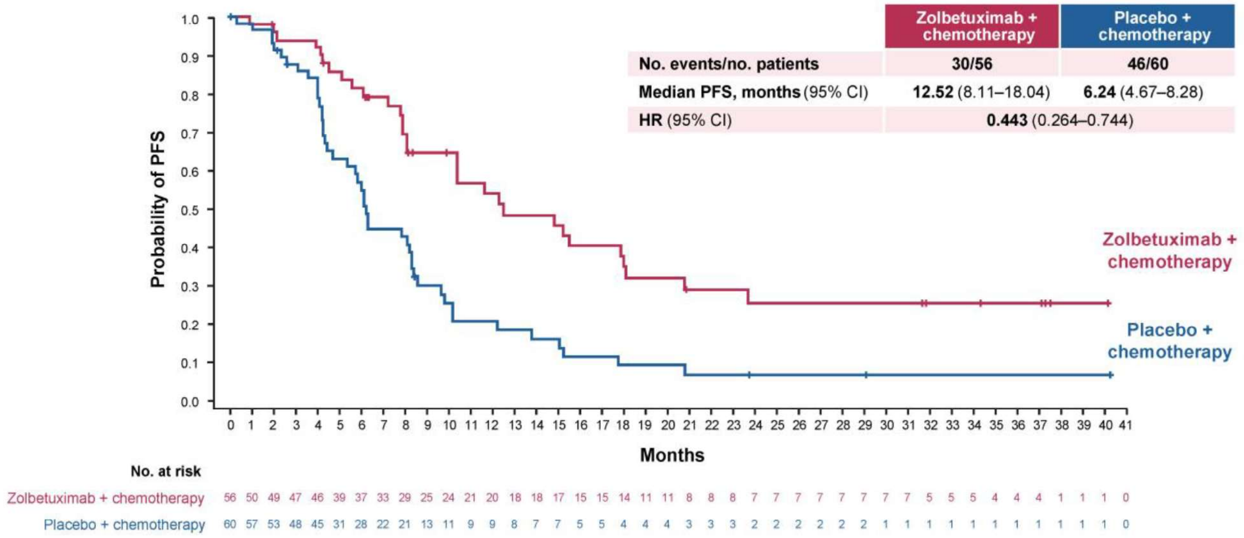

b.

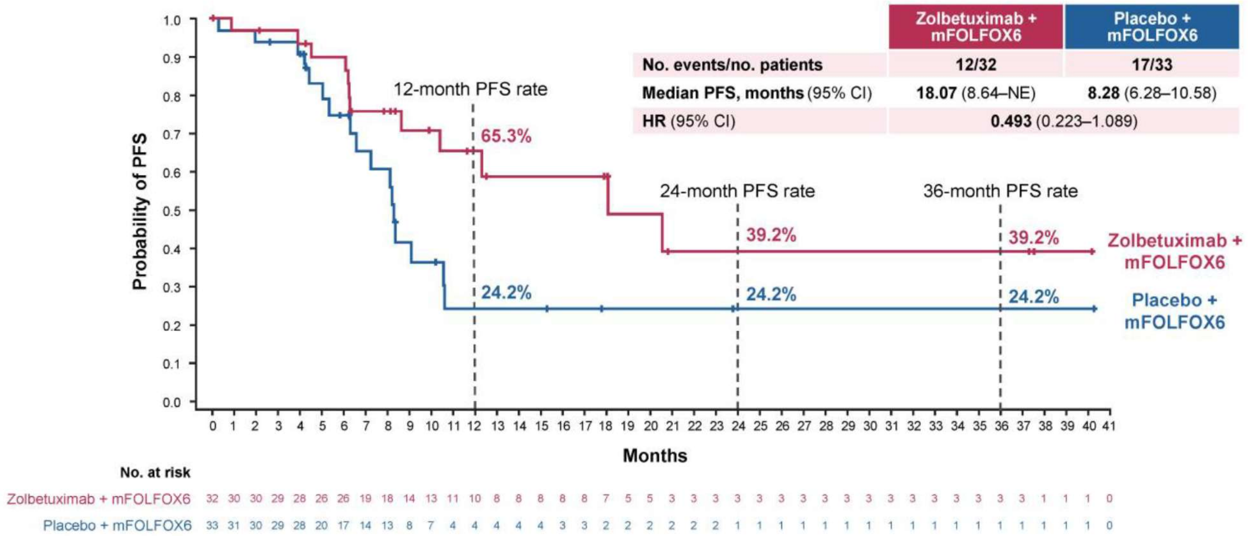

c.

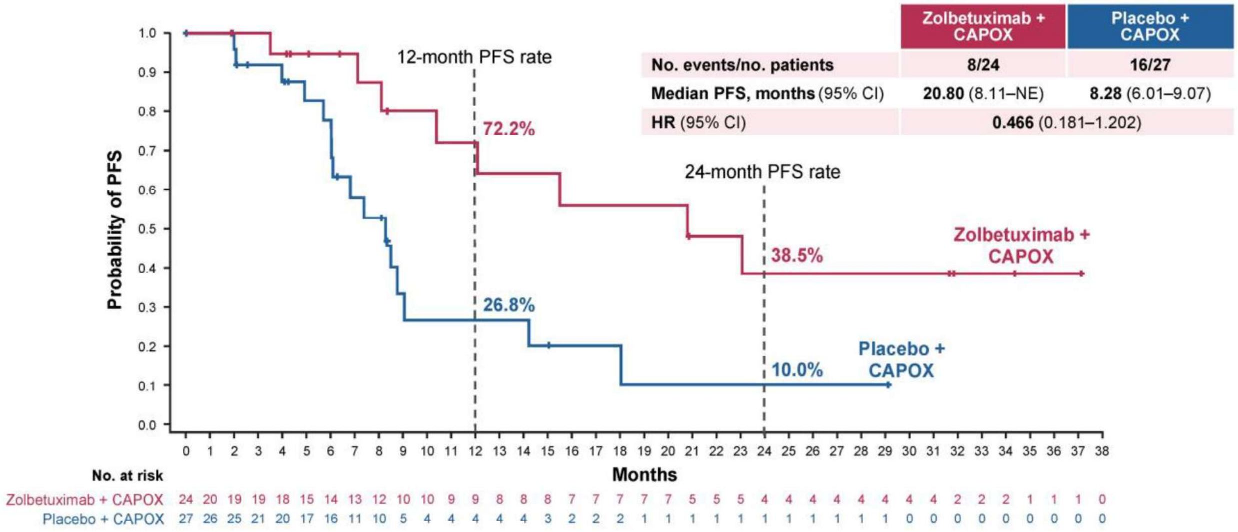

Zolbetuximab + CAPOX
Placebo + CAPOX
No. events/no. patients 8/24 16/27
Median PFS, months (95% CI) 20.80 (8.11–NE) 8.28 (6.01–9.07)
HR (95% CI) 0.466 (0.181–1.202)
12-month PFS rate
24-month PFS rate
72.2%
38.5%
26.8%
10.0%
Zolbetuximab + CAPOX
Placebo + CAPOX
Probability of PFS
1.0
0.9
0.8
0.7
0.6
0.5
0.4
0.3
0.2
0.1
0.0
0 1 2 3 4 5 6 7 8 9 10 11 12 13 14 15 16 17 18 19 20 21 22 23 24 25 26 27 28 29 30 31 32 33 34 35 36 37 38
Months
No. at risk
Zolbetuximab + CAPOX 24 20 19 19 18 15 14 13 12 10 10 9 9 8 8 8 7 7 7 7 7 5 5 5 4 4 4 4 4 4 4 4 2 2 2 1 1 1 0
Placebo + CAPOX 27 26 25 21 20 17 16 11 10 5 4 4 4 4 4 3 2 2 2 1 1 1 1 1 1 1 1 1 1 1 0 0 0 0 0 0 0 0 0

Fig. S2 OS by study.
a) OS in the SPOTLIGHT Japanese subgroup. **b**) OS in the GLOW Japanese subgroup. *CAPOX* capecitabine and oxaliplatin, *CI* confidence interval, *HR* hazard ratio, *mFOLFOX6* modified folinic acid (or levofolinate), fluorouracil, and oxaliplatin, *OS* overall survival

a.

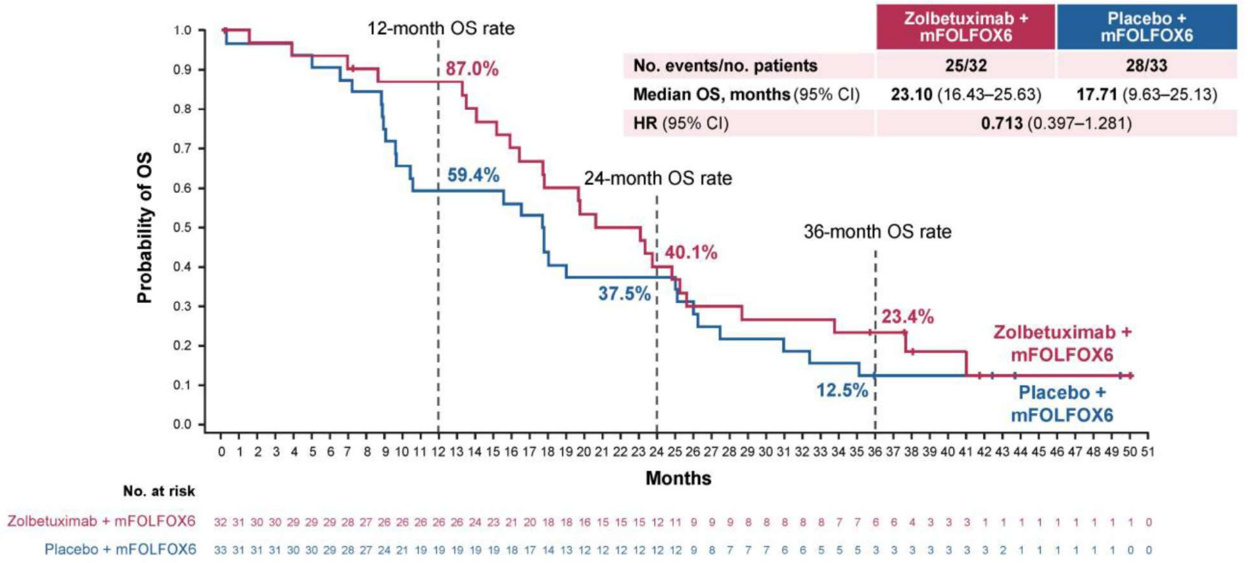

b.

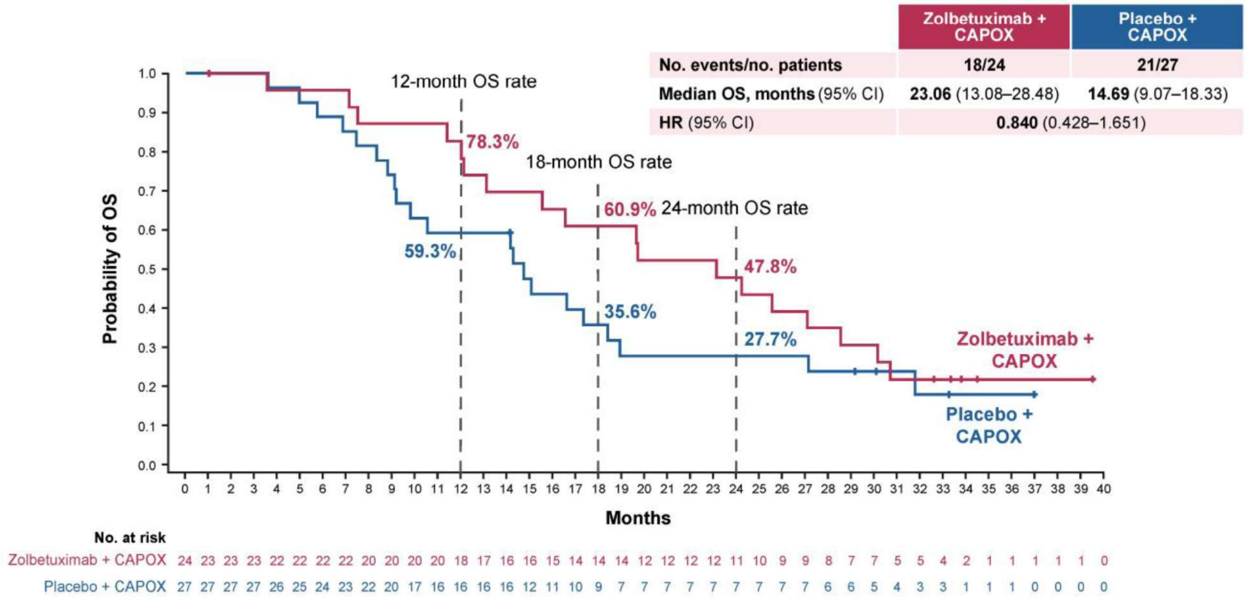

Fig. S3 Waterfall plot for the combined Japanese subgroup.
Best percent change from baseline in tumor size (sum of diameters) by IRC in the full analysis set of the Japanese subgroup. Chemotherapy was either mFOLFOX6 or CAPOX. *CAPOX* capecitabine and oxaliplatin, *IRC* independent review committee, *mFOLFOX6* modified folinic acid (or levofolinate), fluorouracil, and oxaliplatin

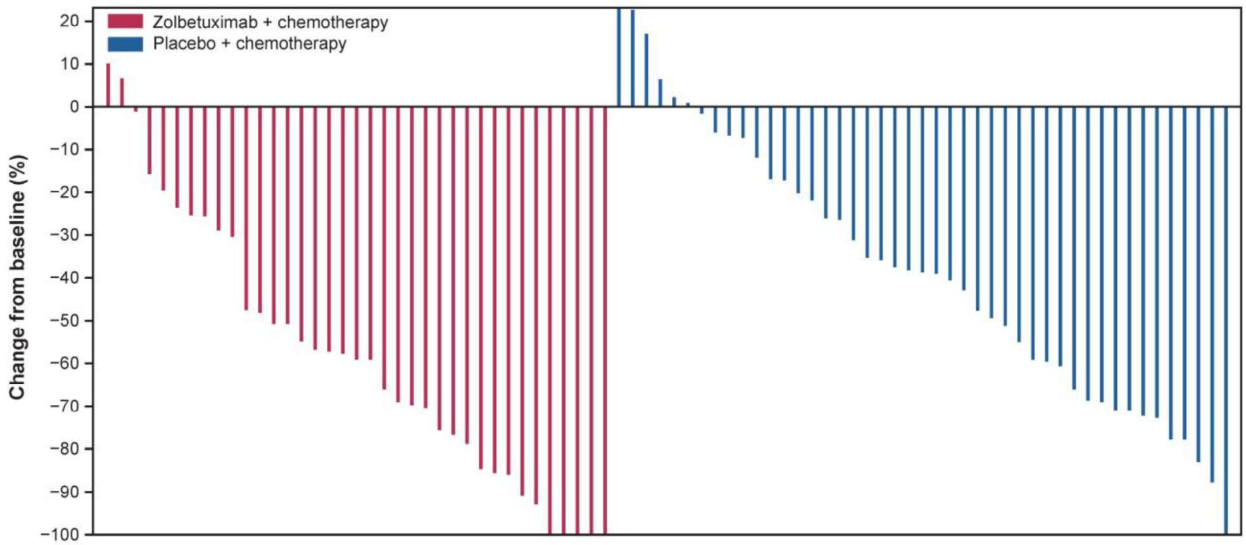

Supplement: Supplementary file 1 — Supplementary Material 1 [file 10120_2026_1738_MOESM1_ESM.pdf]
